# Congenital Anomaly Diagnosis of Typical Cardiac Chest Pain Due to Thebesian Veins Draining Into the Left Ventricular Chamber: A Case Report and Review of Literature

**DOI:** 10.7759/cureus.59002

**Published:** 2024-04-25

**Authors:** Fawaz Mohammed, Zaina Ali Khan, Muna Mohammed, Jeffrey Foley, Mohammad Abdul-Waheed, Muhammad Akbar, Mohammed Kazimuddin

**Affiliations:** 1 Internal Medicine, University of Kentucky College of Medicine, Bowling Green, USA; 2 Internal Medicine, Deccan College of Medical Sciences, Hyderabad, IND; 3 General Surgery, Deccan College of Medical Sciences, Hyderabad, IND; 4 Cardiology, University of Kentucky, Bowling Green, USA

**Keywords:** anginal chest pain, ischemia, coronary steal, congenital, thebesian veins

## Abstract

Thebesian veins are microfistulae that drain the coronary arteries directly into one or more chambers of the heart. Persistence of these anomalous connections into adulthood can lead to shunting of blood away from the myocardium causing typical chest pain symptoms with electrocardiogram changes consistent with ischemia. We describe a case of a 77-year-old female who underwent ischemic evaluation for her symptoms found to have significant Thebesian veins. We also engage in a comprehensive review of the literature finding consistencies in the way these cases are presented in the literature.

## Introduction

The Thebesian venous system is formed by small fistulous passages draining a coronary artery directly into the atrium or ventricle [1]. This venous system is a rare anomaly found on coronary angiography [2]. The majority of patients with these microfistulae remain asymptomatic although some go on to develop ischemia through the phenomenon of coronary steal [3]. First described in 1706 by Vieussens, these primitive venous vessels supply the subendocardial tissue of the myocardium during early embryonic development [4,5]. Interruption during this period leads to the persistence of these venous channels through adulthood creating a pathway where the blood is not able to reach the more finer capillaries of the myocardium. Herein, we report a case of a 77-year-old female with a rare anatomic finding of significant Thebesian veins draining into the left ventricular (LV) chamber presenting with typical anginal symptoms. We also engage in a review of literature of cases published in the literature finding similarities in the way these cases present.

## Case presentation

**Figure 1:**
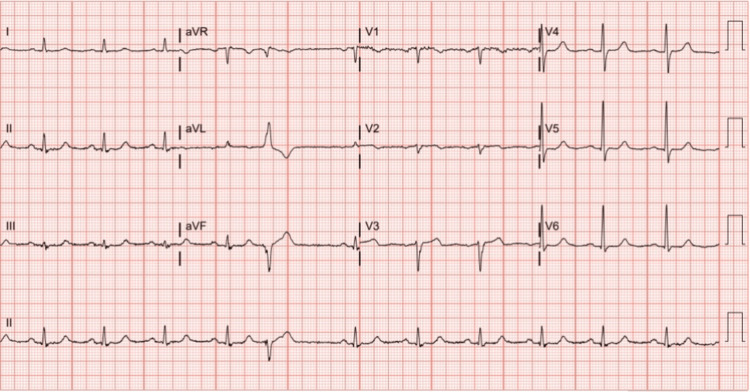
Electrocardiogram finding Electrocardiogram revealing T wave inversions in V1 and V2.

**Figure 2:**
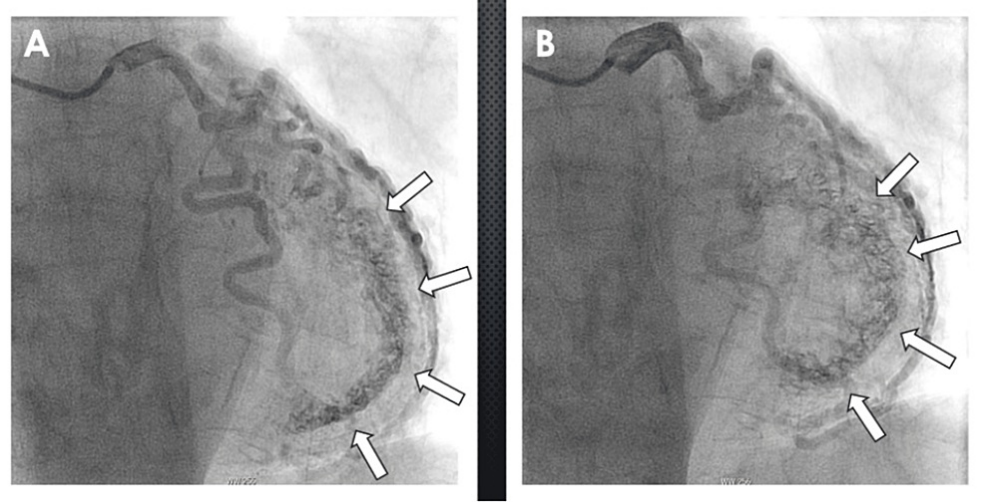
Coronary angiography findings Coronary angiography of the left main coronary artery, left anterior descending artery, and left circumflex artery. (A) The left circumflex artery filling the Thebesian veins through the myocardium, which is outlined by the arrows. (B) Thebesian veins, outlined by the arrows, filling the left ventricular cavity during systole.

A 77-year-old female with a past medical history significant for hypertension, hyperlipidemia, a 50-pack-year smoking history, and a family history of premature coronary artery disease presented in the outpatient setting with complaints of intermittent left-sided chest pain worse with exertion and relieved with rest. Her electrocardiogram (EKG) revealed T wave inversions in the septal leads (Figure 1). A transthoracic echocardiogram revealed an LV ejection fraction of 45-50% with mild global hypokinesis. Given her significant cardiac risk factors and strong family history of coronary artery disease, she was taken to the cardiac catheterization laboratory for a coronary angiogram to rule out ischemic heart disease. On coronary angiography, the dominant right coronary artery and left main were angiographically free of disease. Injection of dye revealed a capillary blush in the LV chamber with the left circumflex artery system providing significant Thebesian veins draining into the LV cavity (Figure 2). She was conservatively managed with aggressive risk factor modification and medical management comprising of aspirin 81 milligrams (mg) daily, carvedilol 6.25 mg twice a day, and atorvastatin 20 mg daily and scheduled to follow-up in the outpatient setting in one month.

## Discussion

Methods

We conducted a comprehensive literature search of all reported cases of Thebesian veins on PubMed, Embase, and Google Scholar between the years 1999 and 2023 using the keyword “Thebesian veins.” We did not enforce any language restriction. A total of 45 cases were retrieved.

Data of Interest

Age, sex, clinical presentation, electrocardiogram (EKG) findings, serum troponin, and coronary angiographic findings were extracted. Mean age and standard deviation were calculated. Serum troponin was categorized as being normal or elevated. EKG descriptions for each case were analyzed and classified as having T wave inversions versus no T wave inversions.

Results

The age range was 52 to 91 years with a mean of 71.9 (SD ±10.7). Of the patients, 31 (71%) were females. Chest pain was the most commonly reported symptom seen in 27 (60%), followed by shortness of breath (11, 24%) and palpitations (5, 11%). Troponin elevation was seen in nine (20%) patients. A total of 18 (40%) cases had T wave inversions in the precordial leads on EKG. The most common fistulous connection identified was between the branches of the left coronary artery (LCA) draining into the left ventricle (LV) seen in 42 (93%) individuals.

Thebesian vessels help drain the blood supply of the myocardium by directly connecting branches of the coronary artery to the atrial and ventricular cavities. These veins, which are part of the lesser cardiac venous system, can rarely present with cardiac chest pain. Symptoms occur when they drain a substantial amount of blood into the chambers of the heart. A coronary fistula occurs when an aberrant connection between a coronary artery and either a vein, another artery, or one of the heart chambers exists [6]. Thebesian veins are an integral part of the congenital cardiovascular system; however, they contribute only minimally to heart circulation in adults [1]. They are rarely extensive enough to cause shunting of the coronary vascular system, meaning the blood supplying the heart takes an alternate abnormal path forming a loop that diverts blood away from the favored capillary network of the myocardium. These aberrant pathways can then cause patients to have symptoms of angina, shortness of breath, or even acute coronary syndrome (ACS) in some cases. ACS tends to occur due to the phenomenon of coronary steal leading to decreased blood supply to the myocardium [7]. An estimated 0.08%-0.3% have a solitary coronary artery draining into a cardiac chamber, while all three major coronary arteries emptying into a cardiac chamber are even rarer [7]. They have clinical implications in selective cases [8].

EKG changes noted in our patient replicated those described in the literature in patients with extensive Thebesian veins [1,3,6,7]. The literature also suggests troponin elevation may be present in some but not all patients [1,3,6,9]. Frank et al. have also suggested that when Thebesian veins cause hemodynamic instability they can mimic EKG changes comparable to Wellens syndrome [10].

When extensive, Thebesian veins can be detected through an echocardiogram with Doppler and cardiac MRI. However, they are most reliably diagnosed with coronary angiography. Heart catheterization showed evidence of Thebesian veins emptying into the left ventricular chamber in the majority of published case reports, with our review showing up to 90% of cases having fistulous connections draining into the LV chamber [1,3,6,9].

Medical management comprises beta blockers and nitrate vasodilators [7]. There is a risk of augmenting the coronary steal phenomenon with nitrate therapy, thus making the use of nitrates controversial [1]. The use of renin-angiotensin-aldosterone system blockers has also been mentioned in the literature [11-13]. From a surgical perspective, treatment options are limited to coronary artery bypass grafting or trans-myocardial laser revascularization (TMLR). In addition, managing existing comorbidities and risk factors related to coronary artery disease is crucial. Published case reports are summarized in Table 1.

**Table 1:** Summary of cases of Thebesian veins in the literature AF, atrial fibrillation; CRBB, complete right bundle branch block; DG, diagonal branch; F, female; LAD, left anterior descending artery; LCA, left coronary artery; LVH, left ventricular hypertrophy; LV, left ventricle; LCx, circumflex artery; M, male; PD, posterior descending artery; RCA, right coronary artery; RV, right ventricular; RVR, rapid ventricular rate; SOB, shortness of breath; (-), no information available.

Author, year	Age	Sex	Clinical presentation	Troponin levels	EKG findings	Coronary artery fistulae/angiogram findings
Gangadharamurthy et al. (2021) [14]	60	F	Chest pain, SOB	Normal	Sinus rhythm, no ST changes	RCA and LCA venoluminal ventriculogram
Crawford et al. (2021) [15]	66	M	Chest pain	Normal	ST elevation in the anteroseptal leads	LAD and RCA draining into the RV, LCx into the LV
Stampfli et al. (2020) [16]	59	F	Asymptomatic	Normal	Lateral T wave inversions	Multiple shunts from draining into the LV
Chattopadhyay et al. (2010) [10]	82	M	Chest pain	Elevated	ST-T wave changes	RCA draining into the LV
Jung et al. (2012) [3]	83	F	Chest pain, SOB	Normal	T wave inversions in V2, V3, V4	LAD, LCx, and RCA draining into the LV
Meuwese et al. (2014) [17]	76	F	Asymptomatic	Normal	Ventricular tachycardia	LCA and RCA draining into the LV
Alam et al. (2015) [18]	72	F	Chest pain	Elevated	Anterolateral T wave inversion	LAD and RCA draining into the LV
Boeder et al. (2017) [8]	64	F	Chest pain	Normal	Sinus rhythm	LCA and RCA draining into the LV
Kim (2020) [13]	75	F	Chest pain	Elevated	T wave inversion V3-V6	Microfistulae from the diagonal branch of LAD draining into the LV
Scoglio et al. (2020) [19]	57	F	Type 2 respiratory failure	-	T wave inversion V2-V4	Multiple Thebesian veins draining into the LV
Somasundaram et al. (2006) [20]	57	F	Palpations, SOB, chest pain, presyncope	-	Ectopic P waves	Patent vein of Marshall communicating with Thebesian vein
Potu et al. (2020) [21]	52	F	Chest pain from spontaneous coronary artery dissection	-	-	Incidental finding of LV Thebesian veins on ventriculography
Khoueiry et al. (2014) [22]	65	F	Chest pain	-	T wave inversion in leads V1-V4	LCA and RCA draining into the LV and RV
Mizuguchi et al. (2013) [23]	Case 1: 83	F	Chest pain	Elevated	Non-specific ST-T changes	LAD draining into LV
	Case 2: 79	M	Chest pain	-	AF, ST-segment depression, and negative T-waves in leads V3-6	LAD draining into LV
	Case 3: 88	F	Vomiting	-	Negative T-wave in inferior leads	DG
	Case 4: 83	M	Chest pain	-	Normal	DG
	Case 5: 79	M	Chest pain	-	ST-T elevation in inferior lead	DG
	Case 6: 81	F	Precordial discomfort	-	AF, ST-T depression in V5, V6	DG
	Case 7: 82	F	Precordial discomfort	-	Poor R-wave progression in V1, V2	DG
	Case 8: 72	F	Palpitation	-	Non-specific ST-T change with LVH	DG
	Case 9: 80	M	Chest pain	-	Normal	DG
	Case 10: 90	F	Palpitation	-	AF, ST-T elevation in V5, V6	DG
	Case 11: 81	F	Effort dyspnea	-	AF, non-specific ST-T change with LVH	DG
	Case 12: 70	F	Effort dyspnea	-	Positive U-wave in V2-V6	DG
	Case 13: 86	F	Chest pain	-	Negative T-wave in V2-6	DG
	Case 14: 81	M	Asymptomatic	-	Normal	DG
	Case 15: 78	F	Effort dyspnea	-	CRBB	DG + PD
	Case 16: 73	F	Chest pain	-	Normal	DG
	Case 17: 91	F	Chest pain	-	AF	DG
	Case 18: 66	M	Chest pain	-	Negative T-wave in inferior leads	DG
	Case 19: 70	M	None	-	Negative T-wave in V4-V6	DG
	Case 20: 76	F	Palpitation, precordial discomfort	-	Normal	DG
Bulut et al. (2007) [24]	55	-	Exertional chest pain	-	LVH	LAD and RCA draining into the LV
Jung et al. (2012) [3]	83	F	Chest pain, SOB	Normal	T-wave inversions on lead V2, V3, and V4	LAD, LCx, and RCA draining into the LV
Kim et al. (2020) [13]	75	F	Chest pain	Elevated	T wave inversions V3-V6	Diagonal branch of LAD draining into the LV cavity
Dixit et al. (2021) [25]	67	F	Chest pain, lightheadedness	Elevated	T wave inversions V3-V6	LAD draining into the LV
Moon et al. (2013) [26]	52	F	Chest pain	-	Negative T waves on precordial leads	LCA draining into the LV
Koganti et al. (2011) [27]	62	F	Syncope	-	-	LAD draining into the LV
Xu et al. (2021) [28]	63	F	Chest pain, SOB	Elevated	Inverted T waves in the anteroseptal leads	LCA draining into the LV
Frank et al. (2020) [11]	60s	F	Substernal pressure, SOB	Elevated	Biphasic T waves in V3, T wave inversions in I, aVL, V4-V6, and ST depressions in V4-V6 and aVF	LAD, RCA, and LCx draining into the LV cavity
Karakas et al. (2022) [29]	70	M	Chest pain, SOB	-	Biphasic T waves in V3, V4, V5, V6	LAD, RCA, and LCx draining into the LV cavity
Agarwal et al. (2014) [30]	71	M	Chest pain	-	Normal	LAD and right ventricular branch of the RCA draining into the RV cavity
Weaver et al. (2010) [31]	69	F	SOB	-	Sinus rhythm with left anterior hemiblock	RCA and LAD draining into the LV
Krishnan et al. (2008) [1]	54	M	Chest pain, Palpitations	Elevated	AF with RVR	Left main and RCA draining into the LV

## Conclusions

Our case with a relevant review of the literature adds to the growing body of evidence of Thebesian veins as a cause of myocardial ischemia presenting with typical chest pain signs and symptoms. Further research is required to understand how this anomaly can affect long-term outcomes and how optimal care can be provided to individuals with this rare anomaly.
